# Association Between Problematic Smartphone Use and Temporomandibular Disorder Severity: A Cross-Sectional Study in Puducherry

**DOI:** 10.7759/cureus.108085

**Published:** 2026-05-01

**Authors:** Neil Dominic, Devakumari S, Satish Kumar, Surendran Venkataraman

**Affiliations:** 1 Oral and Maxillofacial Surgery, Indira Gandhi Medical College and Research Institute, Puducherry, IND; 2 Dentistry, Indira Gandhi Medical College and Research Institute, Puducherry, IND; 3 Otolaryngology, Indira Gandhi Medical College and Research Institute, Puducherry, IND; 4 Community Medicine, Indira Gandhi Medical College and Research Institute, Puducherry, IND

**Keywords:** cross-sectional study, problematic smartphone use, sleep quality, smartphone addiction, temporomandibular disorders

## Abstract

Background: Problematic smartphone use (PSU) has been associated with musculoskeletal strain, psychological distress, sleep disturbances, and reduced physical activity. Temporomandibular disorders (TMD) are multifactorial conditions that these factors may influence. However, the relationship between PSU and TMD severity remains unclear. Materials and methods: A cross-sectional analytical study was conducted among 233 adult smartphone users at Indira Gandhi Medical College and Research Institute, Puducherry, between April 2025 and September 2025. Participants were recruited via convenience sampling from patients attending the dental and ENT outpatient departments. Data were collected using validated instruments, including the Smartphone Addiction Scale, Fonseca Anamnestic Index, Patient Health Questionnaire, Pittsburgh Sleep Quality Index, and International Physical Activity Questionnaire. Participants were categorized based on PSU, TMD severity, psychological distress, sleep quality, and physical activity levels. Associations were assessed using chi-square tests, and binary logistic regression analysis was performed to identify predictors of TMD severity. Results: PSU was observed in 73% of participants. Mild TMD was present in 62.2% of participants, while 36.1% had moderate TMD. No significant association was found between PSU and TMD severity (p = 0.53). Logistic regression analysis demonstrated that PSU, psychological distress, sleep quality, and physical activity were not significant predictors of TMD severity. Sex was the only variable significantly associated with TMD severity (OR = 0.432, p = 0.006). Conclusions: PSU was not significantly associated with TMD severity. These findings support the multifactorial nature of TMD and suggest that smartphone use alone may not independently influence disease severity.

## Introduction

The widespread adoption of smartphones has dramatically transformed modern communication, education, entertainment, and professional activities. With increasing accessibility and affordability, smartphones have become an integral component of daily life across all age groups [1]. Despite their numerous advantages, concerns have emerged regarding excessive smartphone use and its potential adverse effects on physical and psychological health [2,3].

Problematic smartphone use (PSU) refers to a behavioral pattern characterized by excessive, uncontrolled, and compulsive smartphone engagement that interferes with daily functioning [2]. Previous studies have reported an increasing prevalence of PSU worldwide, particularly among young adults and working populations. Response: Thank you for the suggestion. The repetitive text has been consolidated into a single, concise sentence as recommended. Such postural and biomechanical stresses may also influence the temporomandibular joint (TMJ) and associated musculature [4].

Temporomandibular disorders (TMD) represent a group of conditions affecting the TMJ, masticatory muscles, and surrounding structures. Common symptoms include jaw pain, clicking sounds, limited mouth opening, and functional impairment. TMD is considered a multifactorial disorder involving biological, psychological, and behavioral factors. Psychological stress, sleep disturbances, parafunctional habits, and musculoskeletal dysfunction have been identified as important contributors to TMD [5].

Recent research has suggested that excessive smartphone use may indirectly influence TMD through several mechanisms. These mechanisms include prolonged poor posture, increased muscle tension in the cervical and craniofacial regions, psychological distress, and disrupted sleep patterns due to prolonged screen exposure and blue-light emission [6].

However, evidence regarding the relationship between PSU and TMD remains limited and inconsistent. Some studies have reported associations between smartphone overuse and musculoskeletal symptoms in the neck and shoulder regions, while others have suggested potential links with temporomandibular symptoms [7]. Nevertheless, the extent to which smartphone use contributes to TMD severity remains unclear.

Therefore, the present study aimed to evaluate the association between PSU and TMD severity among adult patients attending a tertiary healthcare center in Puducherry, while simultaneously exploring the association of psychological distress, sleep quality, and physical activity with TMD severity.

## Materials and methods

Study design and setting

A cross-sectional analytical study was conducted at Indira Gandhi Medical College and Research Institute, Puducherry, between April 2025 and September 2025.

Study participants

Participants were approached consecutively among eligible attendees in the dental and ENT outpatient departments during the study period using convenience sampling. Adults aged 18 years and above who were smartphone users and willing to provide written informed consent were included in the study. Individuals unwilling to participate, those unable to complete the questionnaire reliably, and those with incomplete questionnaire responses were excluded from the analysis.

Sample size

The sample size was calculated using the Fleiss formula for comparing proportions [8], implemented in the OpenEpi software (Dean AG, Sullivan KM, Soe MM. OpenEpi: Open Source Epidemiologic Statistics for Public Health. www.OpenEpi.com) [9]. Based on prior studies reporting a high prevalence of PSU among adult populations (30-80%) [10] and its association with TMD and musculoskeletal outcomes [11], an expected proportion of approximately 70% was considered. With a 95% confidence level and 80% power, the minimum required sample size was estimated at 196 participants. To account for potential non-response, the sample size was increased by 15%, resulting in a final target of 230 participants. A total of 233 participants were included in the study.

Data collection tools

Data were collected using a structured questionnaire comprising sociodemographic details and validated instruments assessing smartphone use, TMD symptoms, psychological distress, sleep quality, and physical activity. The following standardized instruments were used:

Smartphone Addiction Scale (SAS)

This scale is used to assess PSU. Higher scores indicate greater levels of smartphone addiction. Participants were categorized into PSU present or absent based on predefined cutoffs [12].

Fonseca Anamnestic Index (FAI)

This index is used to assess the severity of TMD. Based on the total score, participants were categorized into no TMD, mild TMD, or moderate TMD [13]. TMD severity was assessed using the FAI as a self-reported screening instrument; no additional clinical examination was used for diagnostic confirmation.

Patient Health Questionnaire (PHQ)

This questionnaire is used to assess psychological distress. Scores were categorized as absent or present distress based on standard thresholds [14].

Pittsburgh Sleep Quality Index (PSQI)

This index is used to evaluate sleep quality. Participants were categorized as having good or poor sleep quality based on validated scoring criteria [15].

International Physical Activity Questionnaire (IPAQ)

This questionnaire is used to assess physical activity levels. Participants were classified into low, moderate, or high physical activity categories based on MET scores [16].

Study variables

The primary independent variable was PSU. The primary outcome variable was TMD severity. Secondary variables included psychological distress, sleep quality, physical activity, age, sex, education, income, and smartphone usage patterns.

Data collection procedure

Participants were approached consecutively, informed of the study's purpose, and enrolled after providing written informed consent. Questionnaires were administered either through self-administration under supervision or direct interviews. They were administered in English or explained in the local language (Tamil) when required to ensure participant comprehension. All responses were recorded and verified for completeness.

Statistical analysis

Statistical analysis was performed using SPSS Statistics version 27.0 (IBM Corp. Released 2020. IBM SPSS Statistics for Windows, Version 27.0. Armonk, NY: IBM Corp.) [17]. Continuous variables were expressed as mean ± standard deviation, and categorical variables as frequencies and percentages.

Associations between categorical variables were assessed using the chi-square test or Fisher’s exact test where appropriate. Binary logistic regression analysis was performed to evaluate predictors of TMD severity. Both unadjusted and adjusted models were constructed, including variables such as age, sex, psychological distress, sleep quality, physical activity, and smartphone usage characteristics. For binary logistic regression, TMD severity was dichotomized as non-moderate TMD (no TMD and mild TMD) versus moderate TMD. Because the no-TMD group was very small (n = 4), it was combined with the mild TMD category for regression analysis.

A p-value of less than 0.05 was considered statistically significant.

Ethical approval

Ethical approval for this study was obtained from the Institutional Ethics Committee of Indira Gandhi Medical College and Research Institute (approval number: 864/IEC-45/IGMC&RI/PP-45/2025). Written informed consent was obtained from all participants prior to enrollment.

## Results

A total of 233 participants were included in the study. The mean age was 38.23 ± 11.82 years. Among them, 58.8% were male and 41.2% were female. Most participants had secondary (36.1%) or graduate-level education (36.1%). A majority were single (55.4%). Regarding income, 35.6% reported ₹10,000-₹30,000, and 27.0% reported ₹30,001-₹50,000. Smartphone use for more than five years was reported by 41.6%, while 38.6% used smartphones for four to six hours daily (Table 1).

**Table 1:** Sociodemographic characteristics of study participants (N = 233) Income is reported in Indian Rupees (₹). SD: standard deviation

Variable	Category	n (%)
Age (years)	Mean ± SD	38.23 ± 11.82
Gender	Male	137 (58.8)
	Female	96 (41.2)
Education	Primary	27 (11.6)
	Secondary	84 (36.1)
	Graduate	84 (36.1)
	Postgraduate	38 (16.3)
Marital Status	Single	129 (55.4)
	Married	104 (44.6)
Income	<10,000	55 (23.6)
	10,000-30,000	83 (35.6)
	30,001-50,000	63 (27.0)
	>50,000	32 (13.7)
Smartphone use years	<1	10 (4.3)
	1-3	61 (26.2)
	3-5	65 (27.9)
	>5	97 (41.6)
Daily use hours	<2	24 (10.3)
	2-4	70 (30.0)
	4-6	90 (38.6)
	>6	49 (21.0)

Distribution of clinical and behavioral scores

The mean SAS score was 34.40 ± 5.36. The mean FAI score was 38.43 ± 11.74. The mean PHQ score was 6.11 ± 2.25. The mean sleep score was 3.35 ± 1.61, and the MET score was 1378.94 ± 766.26 (Table 2).

**Table 2:** Descriptive statistics of clinical and behavioral measures among study participants (N = 233) SAS: Smartphone Addiction Scale, FAI: Fonseca Anamnestic Index, PHQ: Patient Health Questionnaire, MET: Metabolic Equivalent of Task, SD: standard deviation

Variable	N	Mean	SD	Minimum	Maximum
SAS total	233	34.40	5.36	20	48
FAI total	233	38.43	11.74	10	65
PHQ total	233	6.11	2.25	1	12
Sleep score	233	3.35	1.61	0	8
Total MET	233	1378.94	766.26	108.40	3805.70

Prevalence of PSU and TMD severity

PSU was observed in 170 participants (73%), while 63 (27%) did not exhibit PSU. Mild TMD was present in 145 participants (62.2%), moderate TMD in 84 (36.1%), and no TMD in 4 (1.7%). Psychological distress was present in 62.2% of participants, poor sleep in 11.6%, and low physical activity in 14.2% (Table 3).

**Table 3:** Prevalence of PSU, TMD severity, and related factors among participants (N = 233) PSU: problematic smartphone use, TMD: temporomandibular disorder

Variable	Category	n (%)
PSU	Absent	63 (27)
	Present	170 (73)
TMD severity	No TMD	4 (1.7)
	Mild	145 (62.2)
	Moderate	84 (36.1)
Distress	Absent	88 (37.8)
	Present	145 (62.2)
Poor sleep	No	206 (88.4)
	Yes	27 (11.6)
Physical activity	Low	33 (14.2)
	Moderate	189 (81.1)
	High	11 (4.7)

Association of PSU and other factors with TMD severity

PSU was more frequent among individuals with moderate TMD; however, the association was not statistically significant (p = 0.53). Psychological distress, sleep quality, and physical activity were also not significantly associated with TMD severity (p > 0.05) (Table 4).

**Table 4:** Association between PSU and selected factors with TMD severity (N = 233) Chi-square test was used to assess associations. PSU: problematic smartphone use, TMD: temporomandibular disorder

Variable	Categories	Severity of TMD				
		No TMD	Mild	Moderate	Chi-square value	p-value
PSU	Absence	2 (50)	40 (27.6)	21 (25)	1.62	0.53
	Presence	2 (50)	105 (72.4)	63 (75)		
	Total	4 (100)	145 (100)	84 (100)		
Moderate severe distress	Absence	0 (0)	60 (41.4)	28 (33.3)	3.93	0.14
	Presence	4 (100)	85 (58.6)	56 (66.7)		
	Total	4 (100)	145 (100)	84 (100)		
Poor sleep quality	Absence	3 (75)	129 (89)	74 (88.1)	0.75	0.68
	Presence	1 (25)	16 (11)	10 (11.9)		
	Total	4 (100)	145 (100)	84 (100)		
Physical activity level	Low	1 (25)	24 (16.6)	8 (9.5)	4.82	0.30
	Moderate	3 (75)	112 (77.2)	74 (88.1)		
	High	0 (0)	9 (6.2)	2 (2.4)		
	Total	4 (100)	145 (100)	84 (100)		

Predictors of TMD severity: logistic regression analysis

In the unadjusted model, PSU was not significantly associated with TMD severity (OR = 1.16; 95% CI: 0.62-2.15; p = 0.63). Poor sleep, physical activity, psychological distress, and daily smartphone use were also not significant predictors (Table 5).

**Table 5:** Unadjusted logistic regression analysis of predictors of TMD severity OR: odds ratio, CI: confidence interval

Predictor	B	SE	OR	95% CI	p-value
PSU	0.14	0.31	1.16	0.62-2.15	0.63
Poor sleep	0.06	0.42	1.06	0.46-2.45	0.88
Physical activity	0.15	0.33	1.17	0.63-2.23	0.63
Distress	0.26	0.29	1.30	0.73-2.30	0.35
Daily use hours	-0.17	0.15	0.84	0.62-1.13	0.26

After adjustment for age and sex, PSU remained non-significant (OR = 1.16; 95% CI: 0.618-2.179; p = 0.645). Sex showed a statistically significant association with TMD severity (OR = 0.443; 95% CI: 0.245-0.800; p = 0.007), while age was not significant (Table 6).

**Table 6:** Binary logistic regression analysis of factors associated with TMD severity adjusted for age and sex OR: odds ratio, CI: confidence interval, PSU: problematic smartphone use, TMD: temporomandibular disorder

Predictor	B	SE	OR	95% CI	p-value
PSU	0.148	0.322	1.16	0.618-2.179	0.645
Poor sleep	0.232	0.443	1.26	0.529-3.002	0.601
Physical activity	0.126	0.335	1.135	0.589-2.187	0.706
Distress	0.315	0.299	1.37	0.763-2.461	0.292
Age	-0.013	0.012	0.988	0.964-1.011	0.299
Sex	-0.815	0.302	0.443	0.245-0.800	0.007

In the fully adjusted model, PSU remained non-significant (OR = 1.192; 95% CI: 0.632-2.249; p = 0.587). Among all variables, only sex, female participants showed significantly higher odds of moderate TMD severity compared with males (OR = 0.432, 95% CI: 0.238-0.784; p = 0.006). Other variables, including sleep, physical activity, distress, education, marital status, income, and smartphone usage patterns, were not statistically significant (Table 7).

**Table 7:** Fully adjusted binary logistic regression analysis of predictors of TMD severity OR: odds ratio, CI: confidence interval, PSU: problematic smartphone use, TMD: temporomandibular disorder

Predictor	B	SE	OR	95% CI	p-value
PSU	0.176	0.324	1.192	0.632-2.249	0.587
Poor sleep	0.211	0.445	1.235	0.516-2.954	0.636
Physical activity	0.114	0.339	1.121	0.577-2.179	0.736
Distress	0.318	0.301	1.375	0.762-2.480	0.290
Age	-0.012	0.012	0.988	0.965-1.012	0.328
Sex	-0.840	0.304	0.432	0.238-0.784	0.006
Education	-0.024	0.158	0.977	0.717-1.331	0.880
Marital status	-0.105	0.290	0.901	0.510-1.589	0.718
Income	0.160	0.146	1.174	0.882-1.561	0.271
Smartphone use years	0.121	0.156	1.129	0.832-1.531	0.437
Daily use hours	-0.136	0.158	0.873	0.641-1.189	0.387

## Discussion

The present study evaluated the association between PSU and TMD severity among adults, while also considering psychological distress, sleep quality, and physical activity. Although a high prevalence of PSU (73%) was observed, no statistically significant association was found between PSU and TMD severity, suggesting that smartphone use alone may not independently influence TMD severity and supporting the multifactorial etiology of TMD.

The prevalence of PSU in this study is consistent with recent reports among adult and student populations, reflecting increasing dependence on smartphones for communication, entertainment, and occupational activities [2,18]. Excessive smartphone use has been associated with musculoskeletal discomfort, sleep disturbances, and psychological distress [19,20]. Despite plausible biomechanical mechanisms, such as sustained forward head posture and increased cervical muscle tension [4,20], PSU did not independently predict TMD severity in the present study.

TMD is widely recognized as a multifactorial condition involving a complex interplay of biological, psychological, and behavioral factors [21,22]. Established contributors include occlusal disturbances, parafunctional habits, psychological stress, and musculoskeletal dysfunction [23]. Previous studies have suggested that excessive smartphone use may contribute to musculoskeletal discomfort and altered posture, particularly involving the cervical and upper body regions. However, its direct association with TMD remains inconsistent [24].

Previous studies have reported inconsistent findings. Some studies, particularly among younger populations, have demonstrated an association between higher smartphone addiction scores and TMD symptoms, possibly due to prolonged poor posture and altered craniofacial biomechanics [25]. However, variations in study design, population characteristics, and diagnostic criteria may explain these discrepancies [26].

Sleep disturbances and psychological distress have been implicated in TMD through mechanisms involving altered pain perception and increased muscle tension [27,28]. In contrast, the present study did not demonstrate significant associations with TMD severity, which may be attributable to the relatively lower prevalence of poor sleep and the distribution of psychological distress in the study population.

Physical activity, often considered protective against musculoskeletal disorders, was also not significantly associated with TMD severity [25]. This may be due to the relatively homogeneous distribution of activity levels among participants.

Notably, sex emerged as the only significant predictor of TMD severity, with findings consistent with existing literature indicating a higher prevalence of TMD among females [26]. Hormonal influences, differences in pain perception, and psychosocial factors have been proposed as potential explanations.

An important consideration in interpreting these findings is that the musculoskeletal effects of smartphone use may be more pronounced in regions such as the cervical spine and upper back rather than directly affecting the TMJ [20]. Any influence on TMD may therefore be indirect and moderated by other factors such as parafunctional habits and stress-related muscle activity. Additionally, the cross-sectional design captures exposure at a single time point. It may not reflect cumulative or long-term smartphone use patterns, which could partly explain the absence of a significant association observed in this study. The predominance of mild and moderate TMD cases in this sample may also have reduced the ability to detect associations across the full clinical spectrum of disease.

From a clinical perspective, the findings of this study highlight the importance of adopting a comprehensive, multifactorial approach to assessing TMD. Instead, evaluation should include assessment of psychological factors, lifestyle habits, and parafunctional behaviors, which may play a more substantial role in disease progression. Patient education regarding ergonomic practices, appropriate posture during smartphone use, and moderation of screen time may still be beneficial in reducing overall musculoskeletal strain.

From a public health perspective, these findings highlight the importance of promoting awareness regarding healthy screen-time practices, ergonomic posture, and behavioral strategies to reduce musculoskeletal strain associated with prolonged device use. Although PSU was not directly associated with TMD severity, education regarding responsible smartphone use and posture may still have preventive value.

Future longitudinal studies incorporating objective assessments of smartphone use and postural measures may help clarify these relationships. The use of objective measures, such as digital tracking of smartphone usage and biomechanical assessment of posture, may provide more accurate insights. Additionally, exploring the interactions among behavioral, psychological, and biomechanical factors using advanced analytical models could further clarify the underlying mechanisms linking technology use and musculoskeletal health.

Strengths and limitations

This study has several strengths, including the simultaneous evaluation of multiple behavioral and psychological factors using validated instruments and the use of regression analysis to adjust for potential confounders.

However, the cross-sectional design limits causal inference. Additionally, reliance on self-reported data may introduce recall and reporting bias. The use of convenience sampling from outpatient attendees may have introduced selection bias, and the hospital-based study population may limit generalizability to broader community populations. Furthermore, questionnaire-based assessments may not fully capture objective smartphone usage patterns or detailed postural behaviors, which could influence the observed associations. The study also did not account for concurrent use of other digital devices or occupational and educational factors that may influence smartphone exposure, potentially representing unmeasured confounding.

## Conclusions

PSU was not significantly associated with TMD severity after adjustment for confounders. These findings support the multifactorial nature of TMD and suggest that smartphone use alone may not independently predict disease severity. Clinicians should adopt a comprehensive approach incorporating psychological, behavioral, and ergonomic factors in the assessment and management of TMD. Patient education regarding appropriate smartphone use, posture, and lifestyle modifications may help reduce musculoskeletal strain.
